# Predicting Postpartum Hemorrhage Using Clinical Features Extracted With Large Language Models

**DOI:** 10.1097/og9.0000000000000128

**Published:** 2025-10-16

**Authors:** Elizabeth G. Woo, Israel Zighelboim, Tyler Gifford, Joseph G. Bell, Hannah Milthorpe, Emily Alsentzer, Ryan E. Longman, Jorge E. Tolosa, Brett K. Beaulieu-Jones

**Affiliations:** Center for Computational Medicine and Clinical AI, Department of Medicine, and the Section of Ultrasound, Genetics, and the Fetal Neonatal Care Center, Department of Obstetrics and Gynecology, University of Chicago, Chicago, Illinois; the Department of Obstetrics and Gynecology and the Division of Gynecologic Oncology, St. Luke's Cancer Center, St. Luke's University Health Network, Bethlehem, Pennsylvania; the Department of Biomedical Data Science, Stanford University, Stanford, California; and Maternal Fetal Medicine, Oregon Health & Science University, Portland, Oregon.

## Abstract

**OBJECTIVE:**

To evaluate whether large language models (LLMs) applied to prenatal clinical notes can predict postpartum hemorrhage (PPH) before the onset of labor and to compare model performance across outcome definitions, including a novel intervention-based definition.

**METHODS:**

We conducted a retrospective cohort study within a large regional health network. Two outcome definitions for PPH were used: 1) estimated or quantitative blood loss (EBL–QBL) extracted from clinical notes; and 2) a clinical intervention–based PPH definition (cPPH) designed to capture significant hemorrhage requiring intervention, including transfusion, uterotonics, Bakri balloon, or hysterectomy. We evaluated three PPH prediction pipelines: 1) structured data only—supervised machine learning that used structured electronic medical record data; 2) LLM-direct—direct prediction that used a fine-tuned LLM applied to clinical notes; and 3) LLM-extract—interpretable models that used LLM-extracted features combined with structured data. Model performance was evaluated using an area under the receiver operating characteristic curve (AUROC) on a temporally held-out test set.

**RESULTS:**

Among 19,992 deliveries, 1,156 patients (5.8%) met the EBL–QBL definition of PPH, 321 (1.6%) met the cPPH definition, and 309 (1.5%) met both definitions. The LLM-based direct prediction model achieved the highest AUROC for both PPH definitions (AUROC 0.79–0.80), followed by interpretable models that combined LLM-extracted features with structured data (AUROC 0.76–0.78). Models that used only structured data had the lowest AUROC (0.65–0.71). The LLM-extracted features approach identified 47 significant predictors, including established risk factors such as multiple gestation and previous cesarean delivery.

**CONCLUSION:**

These findings highlight the potential of LLM-based approaches to improve PPH risk stratification beyond structured data alone, with the feature extraction method offering a promising balance between predictive performance and clinical utility. Eventual integration of these methods into clinical workflows could improve early detection and guide targeted preventive interventions.

## Abstract 2

Feature extraction from clinical notes using large language models can be used to predict postpartum hemorrhage before the onset of labor, supporting early identification of at-risk patients.

Postpartum hemorrhage (PPH) is a leading cause of maternal morbidity and mortality worldwide.^1^ Timely identification of high-risk patients is critical for prevention, yet existing PPH risk-stratification tools rely on predefined risk factors and show variable performance^2,3^ and clinical utility.^4–9^ Defining PPH, the outcome for these tools, is heterogenous and remains challenging. Traditional definitions based on estimated blood loss (EBL) can be suboptimal due to inaccurate estimates and measurements.^10–12^ Additional definitions have incorporated various clinical signs, including hypovolemia, though there is little consistency among tools.^13^

Recent machine learning approaches improve prediction but typically require intrapartum data and rely solely on structured electronic medical record (EMR) data (ie, laboratory test results, medications, diagnosis codes), limiting their prospective value.^14,15^ However, unstructured clinical notes contain additional context that may enhance early risk identification. Large language models (LLMs) can extract clinically relevant information from notes without requiring additional labeled data.^16–19^ Although LLMs have been used to directly predict clinical outcomes from text,^20^ this approach lacks interpretability and transparency. Alternatively, LLMs can extract manually verifiable intermediate features for downstream predictive modeling.^21^ These LLM-extracted features may capture more nuanced features, such as elements of obstetric history that are missing or inaccurately recorded in structured fields such as International Classification of Diseases codes.

In this study, we evaluated whether LLMs applied to notes could predict PPH, including a clinical intervention–based definition of PPH (cPPH) before labor onset. We compared three approaches: 1) models that used structured data alone, 2) direct LLM-based prediction from notes (LLM-direct), and 3) interpretable models that used LLM-extracted features from notes combined with structured data (LLM-extract). We hypothesized that clinical notes contain information useful for the prediction of PPH that was not traditionally included in predictive models that used structured EMR data and that LLMs that underwent supervised fine-tuning would provide an upper benchmark for potential predictive accuracy but would lack interpretability. Based on this expectation, we aimed to evaluate whether models trained on LLM-extracted information from notes could approach this benchmark while basing predictions on discrete, interpretable features.

## METHODS

St. Luke's University Health Network is a regional health network in Pennsylvania and New Jersey that comprises 12 hospitals and more than 300 outpatient sites. Labor and delivery services across three facilities handle more than 5,000 births annually. We included pregnant patients within St. Luke's University Health Network at 22 weeks of gestation or more, with two or more prenatal obstetric visits and at least one ultrasonogram between 16 and 24 weeks to assess fetal anatomy. These criteria ensured sufficient prenatal information for model predictions. Deliveries occurred between January 7, 2016, and October 25, 2021. This period coincided with the implementation of the clinical data warehouse at St. Luke's University Health Network and the date the data were first pulled for the study. The institutional review board determined the study to be exempt because it uses only deidentified, retrospective data for secondary analysis and does not link the data to any other source.

We obtained deidentified structured data and clinical notes from the EMR for all mothers in the cohort, spanning from the working age of conception (gestational age from first ultrasonogram or last menstrual period if no ultrasonogram was performed before 14 weeks) to 3 days before delivery admission. To simulate prospective deployment, we used a temporal split: deliveries from January 7, 2016, to December 31, 2019 (n=15,399), for model training, and deliveries from January 1, 2020, to October 25, 2021 (n=4,593), for evaluation. This approach evaluates temporal generalizability and reduces the risk of data leakage from random sampling, where similar patients may appear in both sets. Although temporal splits can reflect real-world data set shifts (eg, changes in PPH definitions or clinical practice during the coronavirus disease 2019 [COVID-19] pandemic), such shifts can provide conservative model evaluation. This study used only retrospective data and did not involve real-time predictions or clinician notification.

We used two outcome definitions for PPH. First, the cPPH definition identified clinically significant hemorrhage that required intervention. We used structured data to identify patients who received: 1) transfusion of one or more units of red blood cells, 2) hysterectomy, 3) intrauterine tamponade balloon placement, or 4) use of three or more medications (uterotonics or tranexamic acid). Although cPPH is not a standard clinical term, we introduced this operational definition to capture a subset of patients who received active clinical interventions suggestive of clinically significant hemorrhage, supplementing EBL- or quantitative blood loss (QBL)–based definitions that can be inconsistently measured. Second, the EBL–QBL definition required EBL or QBL of 500 mL or more for vaginal deliveries or 1,000 mL or more for cesarean deliveries, extracted from notes.^22^ Although current American College of Obstetricians & Gynecologists’ guidelines recommend a definition of blood loos of 1,000 mL or more for all deliveries,^23,24^ we used historical thresholds to ensure consistent classification across all deliveries as the changes were being adopted during the study period. Additionally, the study period spanned a time of transition from EBL to QBL documentation, so we used both EBL and QBL to account for the increased adoption of QBL documentation over the study period. When both were present (only in 12 notes), QBL was used. To extract EBL–QBL, we used regular expressions for templated notes and fine-tuned the LLM using cases with unambiguous flowsheet data. The LLM extraction matched manual human reviewers in 250 validation notes.

We fine-tuned an LLM to perform tasks related to PPH prediction. Specifically, we used Meta's open-source Llama-3.1-8B base model, which was pretrained on a wide range of general-domain text. We adapted this model to our clinical tasks through fine-tuning, in which the model is retrained on examples more specific to a use case (ie, clinical notes) so that it can better recognize and interpret relevant content.

**Fig. 1.**
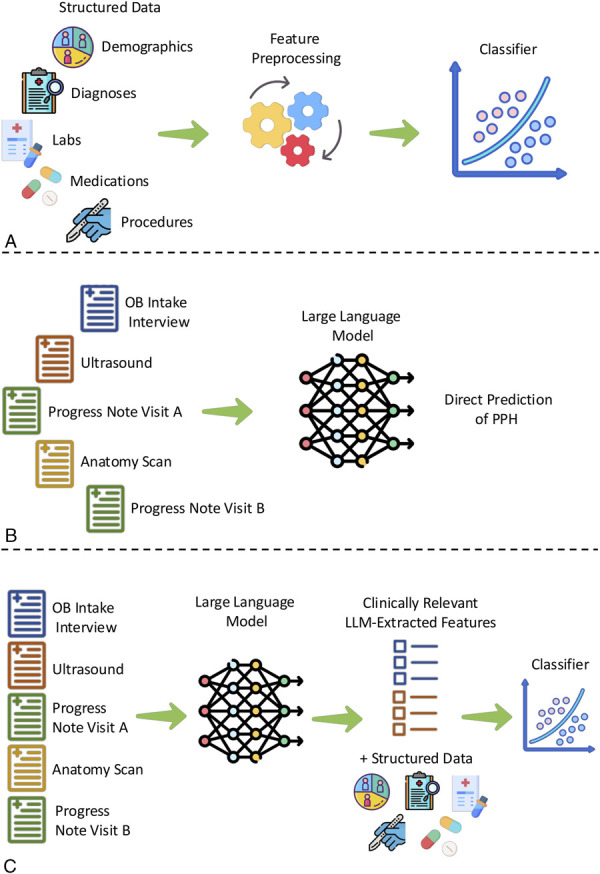
Postpartum hemorrhage (PPH) prediction pipelines. **A**. Predicting PPH with the structured data available in the electronic medical record using logistic regression or XGBoost. **B**. Predicting PPH using a locally pretrained then supervised fine-tuned large language model (LLM, Llama-3.1). **C**. Using a fine-tuned LLM to extract prespecified clinically relevant features as input in addition to the structured data for a supervised classifier (logistic regression and XGBoost).

We performed separate fine-tuning for three tasks: 1) structured data only—extracting EBL–QBL from notes to define PPH (Fig. 1A, preprocessing details and additional details are provided in Appendix 1, available online at http://links.lww.com/AOG/E374); 2) LLM-direct—directly predicting PPH using supervised fine-tuning of an LLM, Llama-3.1 (Fig. 1B) using clinical notes; and 3) LLM-extract—extracting clinical features from notes using a fine-tuned LLM to extract prespecified clinically relevant features (Appendix 2, available online at http://links.lww.com/AOG/E374), which were used along with structured data for PPH prediction (Fig. 1C). Fine-tuning was done using supervised learning on labeled examples (Appendix 1, http://links.lww.com/AOG/E374).

*Woo. Interpretable LLM-Based Prediction of PPH. O&G Open 2025*.

We used knowledge distillation^25^ to develop a feature extraction model that used a teacher–student approach with Llama-3.1-70B-Instruct and Llama-3.1-8B-Instruct. This process improves the model's performance on questions for which the correct answer is unavailable in a note and improves computational performance to make the process feasible on academic hardware. This feature extraction method has been manually validated in prior work for various clinical tasks with high performance.^24^ All extracted features were manually reviewed in 100 charts. These extracted features were combined with the structured data and input into supervised classifiers, including logistic regression and XGBoost. Although structured data were included in both the structured and LLM-extract pipelines, the LLMs only were applied to unstructured clinical notes, which allowed for extraction of features (Appendix 2, http://links.lww.com/AOG/E374) that may not be reliably available in structured data, including detailed obstetric history.

For each prediction model and PPH definition (Fig. 1), we used 10-fold cross-validation to perform parameter selection and hyperparameter tuning on the training data. After all parameter tuning, we selected the top-performing models from cross-validation and applied them to the hold-out evaluation data set. We assessed model performance on the test data using area under the receiving operating curve (AUROC). We calculated 95% CIs and performance comparison using nonparametric bootstrapping of test-set observations (full details in Appendix 1, http://links.lww.com/AOG/E374). Inference on the evaluation data set was performed a single time, and no training or additional parameter tuning was done after inference. To assess feature significance in the LLM-extract logistic regression model, we computed odds ratios for each feature and report the *P*-value adjusted for multiple correction. Features with an adjusted *P*-value less than .05 were considered statistically significant.

## RESULTS

The training data set included 15,399 deliveries that occurred between January 7, 2016, and December 31, 2019. The evaluation data set included 4,593 deliveries between January 1, 2020, and October 25, 2021. The absolute differences in means and proportions between the training and test cohorts in mother's age (train mean 29.83 years, test mean 30.03 years), gestational age (train mean 38.7 weeks, test mean 38.6 weeks), prior live births, and delivery method were small (Table 1). No deliveries met hysterectomy criteria without meeting other, earlier occurring criteria for cPPH. Women with cesarean deliveries averaged more than double the EBL–QBL than women with vaginal deliveries (cesarean: 753.38 mL, vaginal: 345.22 mL; *P*<.001, Appendix 3, available online at http://links.lww.com/AOG/E374). More mothers met the EBL–QBL-based definition than cPPH (Table 1, *P*<.001), with 1,129 mothers (7.3%) in the training data set and 336 (7.3%) mothers in the evaluation data set meeting the EBL–QBL definition and 517 mothers (3.3%) in the training data set and 231 (5.0%) in the evaluation data set meeting the cPPH definition (Appendices 4–6). There were 321 mothers who met the cPPH definition but not the EBL–QBL definition and 1,156 mothers who met the EBL–QBL definition but not the cPPH definition. Mean gestational age at delivery was 37.3–37.5 weeks for mothers who met only the cPPH definition, 38.4–38.7 weeks for those who met only the EBL–QBL definition, and 38.6–38.8 weeks for those who did not meet either definition.

**Table 1.** Demographic Details of the Training and Evaluation Time Periods

Demographic	All Deliveries		Met EBL–QBL Definition Only*		Met cPPH Definition Only^†^		Met Both Definitions		Met Neither Definition	
	Training	Testing	Training	Testing	Training	Testing	Training	Testing	Training	Testing
Total deliveries	15,399	4,593	907	249	211	110	222	87	14,059	4,147
Mother's age at delivery (y)										
Mean	29.83	30.03	30.41	30.96	28.81	30.00	31.21	30.88	29.79	29.94
25th percentile	25.92	26.19	26.72	27.16	24.80	26.29	26.70	26.74	25.91	26.10
50th percentile	29.99	30.20	30.57	31.24	28.38	30.47	31.34	30.35	29.96	30.13
75th percentile	33.71	33.91	34.41	35.57	32.31	34.48	35.75	35.51	33.67	33.83
Gestational age at delivery (d)										
Mean	271.24	270.06	268.70	270.78	261.34	262.75	261.05	264.71	271.71	270.43
25th percentile	268	267	265.50	266.00	256.00	259.50	254.00	261.00	269.00	267.00
50th percentile	275	274	275.00	274.00	272.00	270.00	271.00	274.00	275.00	274.00
75th percentile	280	278	281.00	278.00	278.00	277.50	278.00	278.00	280.00	278.00
Gestational age at delivery (wk)										
Mean	38.7	38.6	38.4	38.7	37.3	37.5	37.3	37.8	38.8	38.6
25th percentile	38.3	38.1	37.9	38.0	36.6	37.1	36.3	37.3	38.4	38.1
50th percentile	39.3	39.1	39.3	39.1	38.9	38.6	38.7	39.1	39.3	39.1
75th percentile	40.0	39.7	40.1	39.7	39.7	39.6	39.7	39.7	40.0	39.7
Prior live births										
0	6,316 (41.0)	1,877 (40.9)	459 (50.6)	129 (51.8)	87 (41.2)	46 (41.8)	104 (46.9)	47 (54.0)	5,463 (38.9)	1,538 (37.1)
1	4,993 (32.4)	1,498 (32.6)	243 (26.7)	63 (25.3)	48 (22.8)	28 (25.5)	42 (20.7)	22 (25.3)	4,697 (33.4)	1,381 (33.3)
2	2,451 (15.9)	739 (16.1)	122 (13.5)	39 (15.7)	35 (16.6)	21 (19.1)	35 (17.1)	8 (9.2)	2,394 (17.0)	756 (18.2)
3 or more	1,639 (10.6)	464 (10.1)	83 (9.2)	19 (7.6)	41 (19.4)	15 (13.6)	34 (15.3)	12 (13.8)	1,505 (10.7)	472 (11.4)
Delivery method										
Vaginal	10,374 (67.4)	3,023 (65.8)	469 (51.7)	92 (37.0)	89 (42.2)	42 (38.2)	100 (45.1)	36 (41.4)	9,710 (69.1)	2,878 (69.4)
Cesarean	4,848 (31.5)	1,432 (31.2)	422 (46.5)	153 (61.5)	115 (54.5)	64 (58.2)	120 (54.1)	49 (56.3)	4,188 (29.8)	1,210 (29.2)
Unspecified or other	177 (1.2)	138 (3.0)	16 (1.8)	4 (1.6)	7 (3.3)	4 (3.6)	2 (0.9)	2 (2.3)	161 (1.2)	59 (1.4)
Transfusion of 1 or more units of red blood cells	321 (2.1)	126 (2.7)	—	—	158 (74.9)	60 (54.5)	163 (73.4)	66 (75.9)	—	—
Intrauterine tamponade balloon	58 (0.4)	20 (0.4)	—	—	11 (5.2)	8 (7.7)	47 (21.2)	12 (13.8)	—	—
3 or more medications (uterotonics or tranexamic acid)	138 (0.9)	85 (1.9)	—	—	53 (25.2)	51 (46.4)	85 (38.3)	34 (39.1)	—	—

EBL, estimated blood loss; QBL, quantitative blood loss; cPPH, clinical intervention–based definition of postpartum hemorrhage.

Data are n (%) unless otherwise specified.

* Estimated or quantitative blood loss of 500 mL or more for vaginal deliveries or 1,000 mL or more for cesarean deliveries.

† Defined as 1) transfusion of 1 or more units of red blood cells, 2) hysterectomy, 3) intrauterine tamponade balloon placement, or 4) use of 3 or more medications (uterotonics or tranexamic acid).

We separately trained three different predictive pipelines for both the EBL–QBL definition of PPH and the cPPH definition. During the training of the models, we performed parameter selection and tuning that used 10-fold cross-validation of the training data (Table 2). The AUROC for the two models was higher in the cPPH definition for all pipelines, which may be, in part due to the variation in the number of mothers matching each definition (Table 1). For both PPH definitions, we observed the highest performance when using the LLM for direct PPH prediction (LLM-direct), with a mean AUROC of 0.844 (95% CI, 0.818–0.869) for cPPH and 0.792 (95% CI, 0.764–0.830) for EBL–QBL. Neither classifier for LLM-extract performed as well as LLM-direct for the cPPH definition (XGBoost: mean AUROC 0.814, 95% CI, 0.791–0.831, *P*<.001; logistic regression: 0.794, 95% CI, 0.770–0.818, *P*<.001) or the EBL–QBL definition (XGBoost: 0.730, 95% CI, 0.718–0.748, *P*<.001; logistic regression: 0.721, 95% CI, 0.704–0.733, *P*<.001). The difference between XGBoost and logistic regression for LLM-extract was not statistically significant for either definition (cPPH: *P*=.210, EBL–QBL: *P*=.398).

**Table 2.** Tenfold Cross-Validation Performance of Classifiers on Training Data (Included Deliveries Between January 1, 2015, and December 31, 2019)

		AUROC	
		cPPH	EBL–QBL
Structured data only	Logistic regression	0.708 (0.683–0.731)	0.656 (0.637–0.667)
	XGBoost	0.730 (0.711–0.746)	0.682 (0.668–0.694)
LLM-direct	LLM	0.844 (0.818–0.869)	0.792 (0.764–0.830)
LLM-extract	Logistic regression	0.794 (0.770–0.818)	0.721 (0.704–0.733)
	XGBoost	0.814 (0.791–0.831)	0.730 (0.718–0.748)

AUROC, area under the receiver operating characteristic curve; cPPH, clinical intervention–based definition of postpartum hemorrhage; EBL, estimated blood loss; QBL, quantitative blood loss; LLM, large language model.

Data are mean (95% CI).

**Fig. 2.**
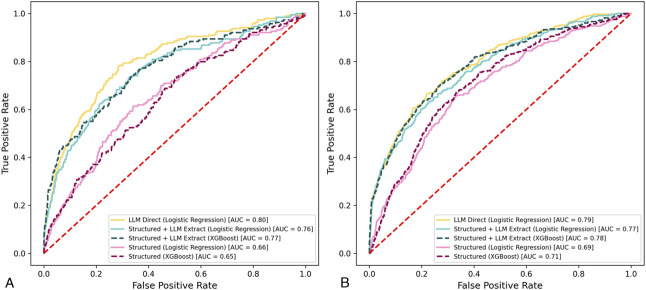
Test set performance for large language model (LLM) and LLM-extracted models. Postpartum hemorrhage (PPH) by clinical intervention (**A**) and PPH by estimated blood loss and quantitative blood loss (**B**). AUC, area under the curve.

We then compared the performance of the direct LLM classifier (LLM-direct) with both XGBoost and logistic regression models using the LLM extracted features (LLM-extract) on the test set. Again, LLM-direct (AUROC 0.79–0.80) slightly outperformed both logistic regression and XGBoost LLM-extract models (AUROC 0.76–0.78; *P*=.002) for both PPH definitions (Fig. 2). Although the LLM-extract models approached performance of LLM-direct, LLM-extract clearly outperformed models that used only structured data (AUROC 0.65–0.71; *P*<.001). Between the models that used only structured data, performance was higher (*P*<.001) with the EBL–QBL definition (logistic regression AUROC 0.69; XGBoost AUROC 0.71) compared with the cPPH definition (logistic regression AUROC 0.66; XGBoost AUROC 0.65). For the LLM-direct and LLM-extract models, performance was not significantly different between the EBL–QBL and cPPH definitions.

*Woo. Interpretable LLM-Based Prediction of PPH. O&G Open 2025*.

We assessed feature significance in the logistic regression model (structured+LLM extract, using the EBL–QBL definition of PPH) and identified 47 significant features (Table 3). For example, multiple gestation (adjusted odds ratio [aOR] 1.28, 95% CI, 1.22–1.35) was a note-extracted feature found in 4.1% (638/15,399) of the deliveries from the training period. Previous cesarean delivery (aOR 1.21, 95% CI, 1.13–1.30; frequency 17.8%) and prior PPH (aOR 1.15, 95% CI, 1.06–1.24) were also note-extracted features that are well-known risk factors for PPH. We also identified significant features from complete blood counts such as abnormal platelet count (aOR, 1.13, 95% CI, 1.02–1.25), high leukocytes (aOR, 1.27, 95% CI, 1.09–1.48), and high immature granulocytes (aOR, 1.16, 95% CI, 1.02–1.31). Maximum platelet count (aOR, 0.79, 95% CI, 0.69–0.91) corresponds with previous work that suggests high prebirth platelet count in the third trimester may be associated with reduced risk of PPH.^26^

**Table 3.** Features Associated With Postpartum Hemorrhage Using Multivariable Logistic Regression Models and Their Frequencies Among Deliveries During the Training Period*

Variable^†^	Source^‡^	Frequency [n (%)]	OR (95% CI)	Adjusted *P*^§^
Obstetric history^‖^	Note	9,659 (62.7)	0.87 (0.78–0.98)	.019
High-risk	Note	4,723 (30.7)	1.09 (1.01–1.18)	.027
Fatigue	Note	4,705 (30.6)	1.09 (1.01–1.17)	.025
Ethnicity: Hispanic	Demographics	3,396 (22.1)	1.08 (1.01–1.15)	.02
Previous cesarean	Note	2,742 (17.8)	1.21 (1.13–1.30)	<.001
Antibiotics used during pregnancy	Medications	1,682 (10.9)	0.90 (0.83–0.98)	.018
Gestational (pregnancy-induced) hypertension without significant proteinuria, 3rd trimester	Diagnoses	1,667 (10.8)	1.09 (1.02–1.15)	.008
Anemia during pregnancy in 3rd trimester	Diagnoses	1,484 (9.6)	1.10 (1.04–1.19)	.033
GDM in pregnancy, unspecified control	Diagnoses	1,434 (9.3)	0.86 (0.75–0.98)	.024
Race: Black	Demographics	1,412 (9.2)	1.08 (1.02–1.14)	.014
Supervision of pregnancy resulting from ART, 2nd trimester	Diagnoses	1,303 (8.5)	1.09 (1.02–1.19)	.049
Shortness of breath	Note	1,293 (8.4)	1.07 (1.01–1.14)	.026
Bleeding during current pregnancy	Note	1,234 (8.0)	0.88 (0.81–0.97)	.006
Anemia, unspecified	Diagnoses	987 (6.4)	1.10 (1.04–1.17)	.002
Maternal care for excessive fetal growth, 3rd trimester, not applicable or unspecified	Diagnoses	987 (6.4)	1.08 (1.02–1.14)	.008
Aspirin	Medications	966 (6.3)	1.08 (1.03–1.14)	.003
Other abnormal findings on antenatal screening of mother	Diagnoses	868 (5.6)	1.06 (1.01–1.12)	.026
Large size for gestational age	Note	738 (4.8)	1.12 (1.04–1.20)	.002
History of leiomyomas or uterine surgery	Note	712 (4.6)	1.09 (1.03–1.15)	.002
Complete placenta previa NOS or without hemorrhage, 2nd trimester	Diagnoses	671 (4.4)	1.08 (1.03–1.14)	.002
Multiple gestation	Note	638 (4.1)	1.28 (1.22–1.35)	<.001
Small size for gestational age	Note	526 (3.4)	0.88 (0.80–0.97)	.013
Placenta previa	Note	337 (2.2)	1.08 (1.03–1.14)	.003
History of hyperemesis gravidarum	Note	265 (1.7)	0.88 (0.80–0.97)	.01
Oligohydramnios	Note	244 (1.6)	1.06 (1.00–1.12)	.05
In vitro fertilization	Note	228 (1.5)	1.11 (1.04–1.19)	.001
Levocetirizine dihydrochloride	Medications	213 (1.4)	1.06 (1.01–1.11)	.025
Nausea and vomiting, intractability of vomiting not specified, unspecified vomiting type	Diagnoses	204 (1.3)	1.12 (1.02–1.23)	.021
Preexisting diabetes	Note	197 (1.3)	1.17 (1.02–1.34)	.029
Hemoglobinopathy	Note	194 (1.3)	1.06 (1.00–1.12)	.032
High-risk postpartum hemorrhage	Note	191 (1.2)	1.08 (1.00–1.16)	.042
History of autoimmune disorders	Note	185 (1.2)	0.91 (0.83–1.00)	.042
Simethicone liquid	Medications	164 (1.1)	1.07 (1.01–1.13)	.019
Type 2 diabetes mellitus	Note	157 (1.0)	0.84 (0.73–0.96)	.012
Prior postpartum hemorrhage	Note	120 (0.8)	1.15 (1.06–1.24)	<.001
High mean corpuscular volume	Note	42 (0.3)	1.05 (1.00–1.10)	.048
No. of obstetric notes during pregnancy	Note	—^¶^	1.16 (1.08–1.24)	<.001
Platelet count (777-3)—maximum value	Laboratory values	—^¶^	0.79 (0.69–0.91)	<.001
Leukocytes (6,690-2)—% abnormal high	Laboratory values	—^¶^	1.27 (1.09–1.48)	.003
Age at pregnancy	Demographics	—^¶^	1.12 (1.03–1.21)	.006
Supervision of other high-risk pregnancies, unspecified trimester	Diagnoses	—^¶^	1.18 (1.04–1.33)	.009
Platelet count (777-3)—% abnormal low	Laboratory values	—^¶^	1.13 (1.02–1.25)	.019
Immature granulocytes (53,115-2)—% abnormal high	Laboratory values	—^¶^	1.16 (1.02–1.31)	.024
Calcium (17,861-6)—% abnormal low	Laboratory values	—^¶^	1.12 (1.01–1.25)	.033
Erythrocyte volume (787-2)—% abnormal low	Laboratory values	—^¶^	1.16 (1.01–1.33)	.034
Creatinine (2,160-0)—% abnormal high	Laboratory values	—^¶^	1.10 (1.01–1.21)	.037

OR, odds ratio; GDM, gestational diabetes mellitus; ART, assisted reproductive technology; NOS, not otherwise specified.

* Data from multivariable logistic regression model using large language model–extracted features and structured data. Postpartum hemorrhage outcome was defined using estimated or quantitative blood loss.

† Prompts used to extract each variable's name are shown in Appendix 2, available online at http://links.lww.com/AOG/E374.

‡ Indicates the origin of the feature. Note denotes large language model–extracted; Laboratory values, Diagnoses, and Medications denote structured electronic medical record input.

§ Adjusted *P* values calculated using multiple comparison correction. Only features with adjusted *P*<.05 are shown.

‖ Obstetric history indicates whether the patient had a prior obstetric history recorded.

¶ Value present for all patients rather than a count (eg, maximum or average laboratory values over the course of a pregnancy).

## DISCUSSION

In this study, we find that using fine-tuned LLMs to directly predict PPH (LLM-direct) achieved higher accuracy than models trained on structured data alone. Training models on structured data combined with discrete clinical features extracted using LLMs recovered much of the performance difference. This indicates that the set of extracted features (Appendix 2, http://links.lww.com/AOG/E374) offer potentially useful predictive value for PPH before the onset of labor.

Although LLM-direct achieved the highest performance, it has several limitations: 1) the time lag between clinical events and note finalization, 2) limited interpretability, and 3) potential behavioral bias. For example, a clinician that believes a patient is at high risk of PPH and would benefit from potential intervention might learn to write notes in a way to ensure the patient receives that intervention.^27^ To address these challenges, we developed LLM-extract, which uses LLMs to extract verifiable clinical features from notes for use in standard prediction models. This extract-then-predict approach offers a practical balance between performance and transparency, performing nearly as well as LLM-direct and clearly outperforming prediction using structured data alone.

Feature analysis identified well-established risk factors for PPH such as multiple gestation, previous cesarean delivery, and prior PPH, supporting the model's ability to capture clinically relevant predictors from notes. This method also identified less commonly considered features associated with increased PPH risk, such as elevated immature granulocyte counts, which has been found to predict the need for massive transfusion in PPH.^28^ This suggests that LLM-guided feature extraction could reveal underappreciated features of PPH risk. Our model also found that bleeding during pregnancy was associated with a slightly lower odds of PPH, despite typically being considered a risk factor. This could reflect closer monitoring among these patients or the model detecting benign bleeding events (ie, spotting) that may carry less risk. Although these findings are hypothesis-generating, they should not be interpreted as definitive associations.

Our goal was not to validate specific predictors, but rather to demonstrate that interpretable features can be automatically extracted from notes at scale and used in transparent models. Most significant features had modest effect sizes, highlighting a limitation of current scoring tools that rely on summation of individual risk factors. Even with structured data extending beyond information used in current clinical risk tools, much of the nuance and context relevant to risk may be better captured in notes. For example, a small proportion of deliveries were noted to have “unspecified” mode of delivery in the structured data. Chart review indicated that these generally represented cases converted to operative and vacuum assisted vaginal delivery, illustrating the value of granularity in clinical notes for clarifying ambiguous structured codes. Replication of this work in additional populations is necessary for rigorous evaluation of features associated with PPH risk.

The strengths of this approach included the exclusion of intrapartum data, enabling risk predictions before labor and avoiding hindsight bias common in many EMR-based models (ie, mode of delivery is often determined during labor, making it challenging to use for prelabor prediction). As such, we did not compare with a model that used only traditional risk factors because prior tools built from these factors often require intrapartum variables unavailable prelabor and have shown relatively lower performance; our focus was instead on whether note-derived features could improve prelabor prediction beyond structured EMR data alone. We also evaluated two PPH definitions: 1) a traditional EBL–QBL-based definition extracted from notes and 2) a novel clinical intervention–based definition that captures clinically significant hemorrhage.

Our study has several limitations. It was conducted at a single health system, limiting both sample size and generalizability. The relatively low incidence and infrequent occurrence of some symptoms reduced our ability to interpret individual features, so our analysis should be considered proof-of-concept rather than definitive identification of risk factors. We also did not assess performance across patient subgroups or model calibration due to the limited sample size but believe both are critical in larger follow-on studies. Importantly, this study explicitly includes patient-reported race and ethnicity as a covariate. Race and ethnicity are sociopolitical constructs, not biological variables, and, in most cases, should not be included in deployed clinical algorithms. The decision to include race in this preliminary discovery study is due to clear evidence that 1) PPH and hemorrhage-related severe maternal morbidity disproportionately affect specific patient subgroups, even after adjustment for measurable comorbidities and obstetric factors^29,30^; 2) there are many relevant unmeasured or undermeasured biologic mediators that correlate with ancestry (eg, blood type) but are missing or difficult to ascertain in EMRs; 3) reporting standards (eg, TRIPOD+AI) explicitly separate model development or discovery from clinical use; and 4) observed inequities motivate, but do not biologize, race (detailed references in Appendix 7, available online at http://links.lww.com/AOG/E374). Using race and ethnicity in discovery may help locate the missing mediators and care-process drivers behind these gaps; it does not imply inherent biological differences. Documentation practices and responses to hemorrhage may vary across subgroups, and future studies should evaluate fairness and potential bias propagation. Although this study was limited to a single institution, we designed our approach to be adaptable to other systems, which would allow us to assess generalizability across diverse hospital settings and populations. For example, institutions that share a common data structure can share code and aggregated results across multiple sites. In the future, federated learning could allow models to be trained across institutions.

This study demonstrates the value of LLMs and unstructured note data to aid in the prediction of PPH before labor onset to support proactive decision-making and care planning before delivery. Although this study serves as a proof of concept of the ability of LLM-extract to identify a subset of key features for prediction models, further steps are required to move toward potential deployment of this approach. This includes validation in larger cohorts and diverse clinical settings, prospective evaluation, and integration into real-time clinical workflows.
